# Molecular imaging of endothelial activation and mineralization in a mouse model of accelerated atherosclerosis

**DOI:** 10.1186/s13550-019-0550-5

**Published:** 2019-08-22

**Authors:** Guillaume Rucher, Lucie Cameliere, Jihene Fendri, Antoine Anfray, Ahmed Abbas, Saïd Kamel, Quentin Dupas, Nicolas Delcroix, Ludovic Berger, Alain Manrique, on behalf of the STOP-AS investigators

**Affiliations:** 1 Normandie Univ, UNICAEN, EA 4650, GIP Cyceron, 14000 Caen, France; 2 Chirurgie Vasculaire, CHU de Caen, Avenue de la Côte de Nacre, 14000 Caen, France; 3 Normandie Univ, UNICAEN, INSERM, UMR-S U1237, Physiopathology and Imaging of Neurological Disorders (PhIND), 14000 Caen, France; 4 Normandie Univ, UNICAEN, EPHE, INSERM, U1077, Neuropsychologie et Imagerie de la Mémoire Humaine, 14000 Caen, France; 5 EA7517, MP3CV, CURS, University of Picardie Jules Verne, Amiens, France; 6 Biochemistry Laboratory, Amiens University Hospital, Amiens, France; 7 CNRS, UMS-3048, GIP Cyceron, Campus Jules Horowitz, 14000 Caen, France; 8 Médecine Nucléaire, CHU de Caen, Avenue de la Côte de Nacre, 14000 Caen, France; 9 GIP Cyceron, Campus Jules Horowitz, Boulevard Henri Becquerel, 5229, 14074 Caen, BP France

**Keywords:** Positrons emission tomography (PET), Magnetic resonance imaging (MRI), Atherosclerosis, Mineralization, Endothelial activation

## Abstract

**Purpose:**

Preclinical imaging of endothelial activation and mineralization using both positron emission tomography (PET) and magnetic resonance (MR) remains scarce.

**Procedures:**

A group of uremic ApoE^−/−^ (Ur), non-uremic ApoE^−/−^ (NUr), and control C57Bl/6 J mice (Ctl) were investigated. Mineralization process was assessed using sodium fluoride ([18F]NaF) PET, and MR imaging combined with intravenous injection of MPIO-αVCAM-1 was used to evaluate endothelial activation. Micro- and macrocalcifications were evaluated by flame atomic absorption spectroscopy and von Kossa staining, respectively.

**Results:**

Ur mice showed an active and sustained mineralization process compared to Ctl mice (*p* = 0.002) using [18F]NaF PET imaging. Calcium plasma level was increased in Ur (2.54 ± 0.09 mM, *n* = 17) compared to NUr and Ctl mice (2.24 ± 0.01, *n* = 22, and 2.14 ± 0.02, *n* = 27, respectively; *p* < 0.0001). Likewise, vascular calcium content was increased in Ur (0.51 ± 0.06 μg Ca^2+^ per milligram of dry weight aorta, *n* = 11) compared to NUr (0.27 ± 0.05, *n* = 9, *p* = 0.013) and Ctl (0.28 ± 0.05, *n* = 11, *p* = 0.014). Ur mice also had a higher inflammatory state using MPIO-αVCAM-1 MR (*p* global = 0.01, post hoc analysis Ur vs. Ctl *p* = 0.003) associated with increased VCAM-1 expression (*p* global = 0.02). Aortic remodeling at the level of the brachiocephalic trunk, brachiocephalic trunk itself, and aortic arch in Ur mice was also demonstrated using MR.

**Conclusions:**

Preclinical molecular imaging allowed in vivo characterization of the early phase of atherosclerosis. [18F]NaF PET showed early and sustained vascular mineralization in uremic ApoE^−/−^ mice. MPIO-αVCAM-1 MR imaging demonstrated aortic endothelial activation, predominantly in segments with vascular remodeling.

## Background

Atherosclerosis is a leading cause of death and disability worldwide and involves several pathophysiological processes including endothelial activation, inflammation, mineralization, and necrosis. Vascular remodeling and the inflammatory state of lesions determine the plaque instability and therefore the risk of subsequent deleterious clinical events (myocardial infarction, stroke). Preliminary clinical studies showed the ability of sodium fluoride ([18F]NaF) positron emission tomography (PET) to highlight active mineralization process within the vascular wall [1, 2]. The deposition of calcium crystals within the plaque during atherosclerosis process is due to phenotypic changes of vascular smooth muscle cells (VSMCs) into osteoblast-like cells [3]. Bone morphogenetic proteins (BMPs), oxydative stress, or changes in pyrophosphate levels are factors that induce osteogenic differentiation in VSMCs. BMP2 is recognized as a mediator of both mineralization and local inflammation in pathologic conditions [4] and was found to be expressed in atherosclerotic lesions [5] as an increased expression of alkaline phosphatase [6].

The [^18^F]-fluorodeoxyglucose (FDG) uptake in arterial wall is proportional to macrophage infiltration and reflects the inflammatory state of atherosclerotic lesions [7]. The estimation of soluble biomarkers such as intracellular adhesion molecule-1 (ICAM-1) or vascular cell adhesion molecule-1 (VCAM-1) also underlines the level of endothelial activation. An upregulation of VCAM-1 expression is a feature of inflammatory conditions [8]. Preclinical magnetic resonance (MR) imaging allows the assessment of deep tissues with a non-ionizing imaging method. Associated with an intravenous injection of micron-sized particles of iron oxide (MPIO) conjugated with specific αVCAM-1 antibodies, MR imaging allows the endothelial activation mapping in mice [9].

In a recent review of the literature [10], the Cardiovascular Study Group of the European Society of Molecular Imaging emphasized the lack of preclinical data on the use of [18F]NaF. Particularly, preclinical investigations may help understand the relationship between early microcalcifications demonstrated by [18F]NaF vascular uptake and both endothelial activation and further vascular remodeling. In the present study, we used both [18F]NaF PET and MPIO-αVCAM-1 MR to assess early endothelial activation and mineralization processes in a mouse model of accelerated atherosclerosis.

## Methods

### Animal model

The institutional animal ethics committee (Comité National de Réflexion Ethique sur l’EXpérimentation Animale CENOMEXA) approved the animal experiments (#3979), and all experiments were performed with European and French regulation. A control group consisted of 31 control C57Bl/6 J mice (Ctl), a group of non-uremic 25 C57Bl/6 J ApoE^−/−^ mice (NUr), and a group of uremic 27 C57Bl/6 J ApoE^−/−^ mice (Ur) were investigated. All animals were male and maintained on a standard chow diet composed by 8.4% fat, 19.3% protein, 72.4% carbohydrates, 0.55% phosphorus, 0.73% calcium, 0.16% magnesium, and 1000 UI/kg vitamin D_3_. We induced chronic renal failure by an electrocoagulation of the right kidney at 8 weeks old, followed 2 weeks later by a contralateral nephrectomy, according to a previously described procedure [11]. Analgesia was induced by injection of buprenorphine (0.05 mg/kg, Buprecase®, Axience, Pantin, France) administered in preoperative and at 9 h interval in postoperative during the first 24 h.

### In vivo PET acquisitions and reconstruction

All acquisitions were performed using a dedicated preclinical PET-CT system (Inveon®, Siemens Healthcare, Erlangen, Germany). At 12 and 16 weeks old, anesthesia was induced with 5% isoflurane gas and maintained with isoflurane 2% gas in a mixed of O_2_ and N_2_O (1:2). Following attenuation CT acquisition, [18F]NaF injection (≈ 18 MBq) was performed intravenously through a tail vein catheter. Fifty minutes after [18F]NaF injection, a 10-min list mode acquisition of emission data was performed (energy and coincidence windows of 350–650 keV and 3.4 ns, respectively). Image series were reconstructed using 3D-ordered subset expectation maximization (3D-OSEM) with 8 iterations and a zoom factor 2 without scatter correction.

Postprocessing was performed using Carimas v2.4 software (Turku PET Centre, Turku, Finland). The [18F]NaF activity was represented with a color scale starting just upon the plasma activity determined drawing a volume of interest over the left ventricle with a maximum set to 200% of the plasma activity measured at the last frame of the data for each examination [12]. An uptake in a region close and above to the heart was considered as a positive aortic [18F]NaF uptake. Then, aortic uptake was semi-quantitatively determined as the ratio of the SUVmax in a VOI drawn over the visible aortic uptake to the SUVmax of vascular background measured in the inferior vena cava. In addition, global cardiac [18F]NaF uptake was also measured in a volume of interest encompassing the whole heart normalized to the same vascular background.

### MR imaging

MR experiments were carried out in a second group of animals using a 7-T magnet (Pharmascan® Bruker, Billerica, USA) with dual respiratory and ECG gating. Telediastolic T2*-weighted MR images were acquired using a multi-slice sequence: field of view 1809 × 939 mm^2^, TR/TE 100/4.25 ms, 12 slices, and voxel 0.1 × 0.1 × 0.15 mm^3^ encompassing the thoracic aorta were performed in 16-week-old mice, with and without the intravenous injection of 200 μL of MPIO-αVCAM-1.

**Fig. 1:**
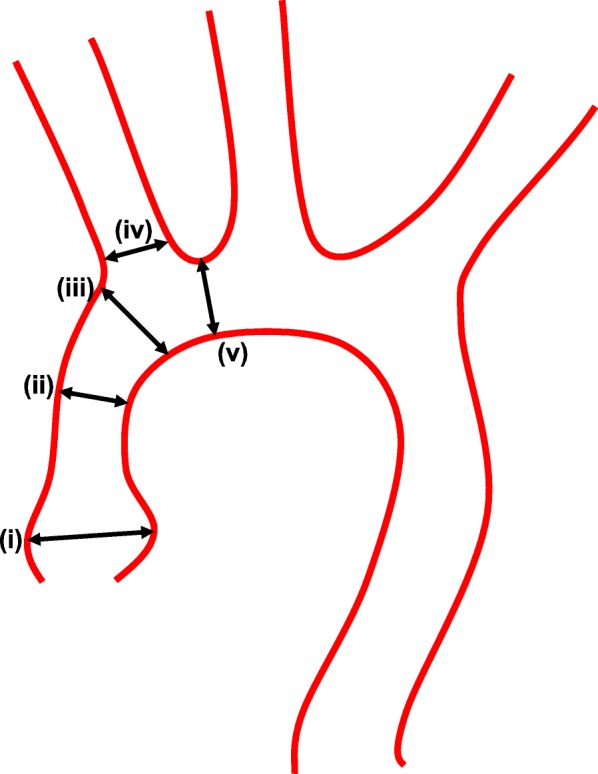
Scheme of aortic diameters measured in MR images. (**i**) The aortic root, (**ii**) the ascending aorta, (**iii**) the aorta at the level of the brachiocephalic trunk, (**iv**) the brachiocephalic trunk, and (**v**) the aortic arch

As previously described [9], microparticles of iron oxide (DynaBeads MyOnes Tosyl Activated, ThermoFisher Scientific, Waltham, USA) were conjugated with antibodies anti-αVCAM-1 (clone A(429), BD BioScience, Franklin Lakes, USA) by incubation at 37 °C for 48 h. MRI images were analyzed using Osirix v.6.5.2 software. MPIO-αVCAM-1 binding results in a T2* signal void in the lumen of the vasculature. Intravascular diameters were measured using non-injected MR images on the following segments: (i) the aortic root, (ii) the ascending aorta, (iii) the aorta at the level of the brachiocephalic trunk, (iv) the brachiocephalic trunk, and (v) the aortic arch (Fig. 1). The diameter measurement was indexed to the animal weight measured the day of the MR acquisition.

### Tissues samples

Mice were sacrificed at 16 weeks old. Blood samples were collected to determine the plasma level of urea and calcium using Beckman-Coulter AU5800 autoanalyzer (Miami, USA). Aortas were harvested from the root to the diaphragm. In 11 Ctl, 9 NUr, and 11 Ur, aortic tissue calcium content was measured by flame atomic absorption spectroscopy (FAAS) (Varian AA240, Varian Inc., Palo Alto, CA). After an overnight bath of aortas in a 0.6 N HCl solution at 4 °C, FAAS was performed in supernatant. Aortas were dried at 37 °C, then weighted, and the vascular calcium concentration was reported on the dry weight of each aorta.

In 6 Ctl, 5 NUr, and 11 Ur, macrocalcifications were assessed using the von Kossa staining. Aortas were dissected under a microscope, perfused with heparinized (50 U/mL) phosphate-buffered saline 5/100 (PBS), and cryomounted in optimal cutting embedding medium (OCT, Thermo Scientific, Waltham, USA). Ten-micrometer-thick slices were collected each 50 μm. Cryosections were placed in 5% silver nitrate solution for 30–60 min then fixed in 5% sodium-thiosulfate solution for 2–3 min. Sections were digitized using ScanScope CS (Leica Byosystems, Wetzlar, Germany,) and tissue segmentation was manually performed using Aperio ImageScope software v12.3 (Leica Biosystem, Wetzlar, Germany). Data were expressed as the relative proportion of stained tissue to total tissue area.

### Western blotting

Harvested aortas were homogenized in homogenization buffer (20 mM Tris HCl, 150 mM NaCl, 1 m EDTA, 1% Triton X-100), then sonicated and centrifuged (20,000 G, 15 min, 4 °C). Total protein concentrations were determined using the Bradford assay (Bio-Rad, Hercules, USA), and protein was solubilized in Laemmli 4× buffer (25 mL TrisSDS, 20 mL glycerol, 4 g SDS, 2 mL βmercaptoethanol, 1 mg Bromophenol Blue, 50 mL H_2_0). This solution was vortexed, and proteins were denatured using thermal cycler GeneAmp PCR system 2700 (Applied Biosystems, Foster City, USA) at 95 °C (3 min). Proteins (50 μg) were separated on a 12% SDS-PAGE gel (86.4 g glycine, 6 g SDS, 18 g TrisBase) and transferred to a nitrocellulose membrane (BioRad, Hercules, USA). Blots were blocked with a blocking solution (non-fat dry milk). Primary antibodies were incubated 12 h at 4 °C with anti-VCAM-1 from Cell Signaling (1:1000, VCAM-1 (D2T4N) Rabbit mAb (Mouse Specific), Ozyme distributor, Saint Quentin en Yvelines, France), anti-OPN-R (1:2500, OPN-R Antibody (24H5L3) ABfinity Rabbit mAb, ThermoFisher Scientific, Waltham, USA), and anti-GAPDH (1:1000, GAPDH Loading Control Antibody (MA5-15738), ThermoFisher Scientific, Waltham, USA). After incubation with appropriate horseradish peroxidase-conjugated secondary antibodies for 1.5 h at room temperature, proteins were visualized by chemiluminescence. Immunoreactive proteins were scanned using Chemidoc XRS (Bio-Rad, Veenendaal, The Netherlands), and intensities were analyzed with ImageJ NIH image processing software (version 1.52a, Bethesda, MD, USA).

### Statistical analysis

Values were expressed as mean ± SEM. Continuous data were compared using the Wilcoxon signed-rank test or Mann-Whitney *U* test when appropriate. Multivariate analysis was performed using logistic regression, with post hoc analysis using Tukey’s HSD test or odds ratio when appropriate. The Kruskal-Wallis test was used for global comparison between three independent animal groups. For proportions, the chi-square test was used to compare differences between groups. Statistical analyses were performed using JMP 11 (SAS Institute, Cary, NC, USA). A *p* value ≤ 0.05 was considered statistically significant.

## Results

### Ex vivo assessment

**Fig. 2:**
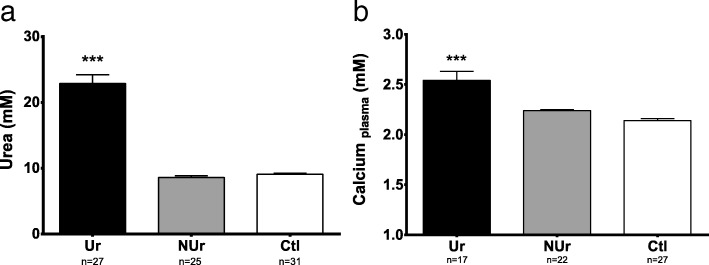
Urea (**a**) and calcium (**b**) plasma level. **a** Urea plasma level (mM) in uremic ApoE^−/−^ mice (Ur, *n* = 27), non-uremic ApoE^−/−^ mice (NUr, *n* = 25), and control Bl6 mice (Ctl, *n* = 31). **b** Calcium plasma level (mM) in uremic ApoE^−/−^ mice (Ur, *n* = 17), non-uremic ApoE^−/−^ mice (NUr, *n* = 22), and control mice (*n* = 27). Data are expressed as mean ± SEM. ***p < 0.0001

**Fig. 3:**
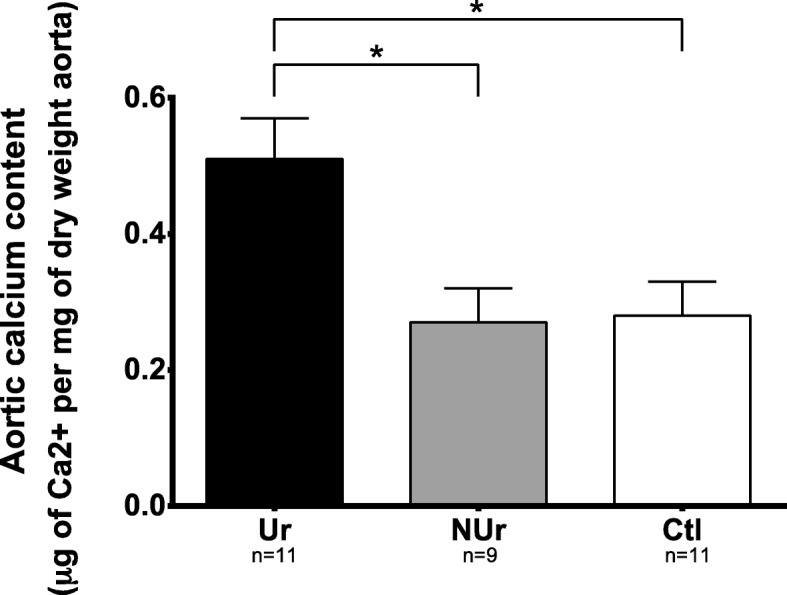
Aortic tissue calcium content. Values of total aortic tissue calcium content in microgram of Ca^2+^ per milligram of dry weight aorta in uremic ApoE^−/−^ mice (Ur, *n* = 11), non-uremic ApoE^−/−^ mice (NUr, *n* = 9), and control Bl6 mice (Ctl, *n* = 11). Data are expressed as mean ± SEM. *p < 0.05

Urea plasma level was significantly increased in Ur (22.87 ± 1.32 mM, *n* = 27) compared to NUr and Ctl mice (respectively 8.62 ± 0.25 mM, *n* = 25, and 9.11 ± 0.15 mM, *n* = 31; *p* < 0.0001; Fig. 2a). The concentration of calcium in plasma was significantly higher in Ur (2.54 ± 0.09 mM, *n* = 17) compared to NUr and Ctl mice (respectively 2.24 ± 0.01 mM, *n* = 22, and 2.14 ± 0.02 mM, *n* = 27; *p* < 0.0001; Fig. 2b). Flame atomic absorption spectroscopy demonstrated a significant increase of vascular calcium in Ur mice (0.51 ± 0.06 μg of Ca^2+^ per milligram of dry weight aorta, *n* = 11) compared to NUr (0.27 ± 0.05 μgCa^2+^/mg, *n* = 9, *p* = 0.013) and Ctl mice (0.28 ± 0.05 μgCa^2+/^mg, *n* = 11 *p* = 0.014; Fig. 3). The von Kossa staining demonstrated a non-significant increase of vascular macrocalcifications in Ur mice (0.48 % ± 0.06, *n* = 11) compared to NUr and Ctl mice (respectively 0.41 ± 0.03, *n* = 5, and 0.43 ± 0.02, *n* = 6, NS).

### PET assessment

**Fig. 4:**
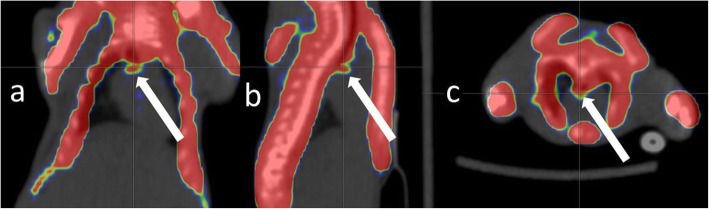
Aortic [18F]NaF uptake in an uremic ApoE^−/−^ mouse. [18F]NaF PET-CT acquired from 50 to 60 min after the injection of 26 MBq of [18F]NaF in a uremic ApoE^−/−^ mouse aged 12 weeks old. **a** Coronal view. **b** Sagittal view. **c** Axial view. White arrow, aortic [18F]NaF uptake

At 12 weeks old, [18F]NaF PET demonstrated an aortic uptake in 8/12 (66%) Ur mice (Fig. 4), 5/9 (55%) NUr mice, and 0/3 (0%) in Ctl (Table 1). However, only Ur mice (4/9, 45%) had an aortic uptake at 16 weeks old (0/4 in NUr mice and 0/4 in Ctl; Table 1). There was a significant impact of animal group on aortic [18F]NaF uptake (*p* = 0.01), and post hoc analysis showed a significant difference between Ur and Ctl mice (*p* = 0.002). A higher proportion of macrocalcifications on the von Kossa staining was noted in mice without aortic uptake compared to mice with aortic [18F]NaF uptake at 16 weeks old (PET− 0.89% ± 0.09 vs. PET+ 0.42% ± 0.07, *p* = 0.028). There was a global effect of the genotype and the age of the animals on cardiac [18F]NaF uptake assessment (global *p* value < 0.05; Table 2). In addition, there was a positive corrrelation between cardiac and aortic [18F]NaF uptake in animals demonstrating positive aortic [18F]NaF uptake (*r* = 0.69, *p* < 0.01).

**Table 1:** Contingency table for PET in detecting [18F]NaF aortic uptake (PET+)

	PET+	PET−	Total (*n*)
12 weeks old			
Ur	8 (66%)	4 (34%)	12
NUr	5 (55%)	4 (45%)	9
Ctl	0 (0%)	3 (100%)	3
16 weeks old			
Ur	4 (45%)	5 (55%)	9
NUr	0 (0%)	4 (100%)	4
Ctl	0 (0%)	4 (100%)	4

The [18F]NaF activity was represented with a color scale starting just upon the plasma activity determined drawing a volume of interest over the left ventricle with a maximum set to 200% of the plasma activity measured at the last frame of the data for each examination [12]. An uptake in a region close and above to the heart was considered as a positive aortic [18F]NaF uptake. *Ur* uremic ApoE^−/−^, *NUr* non-uremic ApoE^−/−^, *Ctl* control mice

**Table 2:** [18F]NaF cardiac and aortic uptake quantification

	Ur	NUr	Ctl
12 weeks old			
Cardiac uptake	2.2 ± 0.1	1.9 ± 0.2	1.8 ± 0.1
Aortic uptake	2.8 ± 0.3	1.9 ± 0.3	ND
16 weeks old			
Cardiac uptake	1.9 ± 0.2	1.5 ± 0.1	2 ± 0.2
Aortic uptake	2.6 ± 0.6	ND	ND

[18F]NaF cardiac uptake was assessed in 12 uremic ApoE^−/−^ (Ur), 9 non-uremic Apo^−/−^, and 3 control mice at 12 weeks old and in 9 Ur, 4 NUr, and 4 Ctl at 16 weeks old. [18F]NaF aortic uptake was assessed in 8 Ur and 5 NUr at 12 weeks old and in 4 Ur at 16 weeks old. Results are expressed in arbitrary units. *ND* not done when there was no visible aortic uptake

### MR assessment

**Fig. 5:**
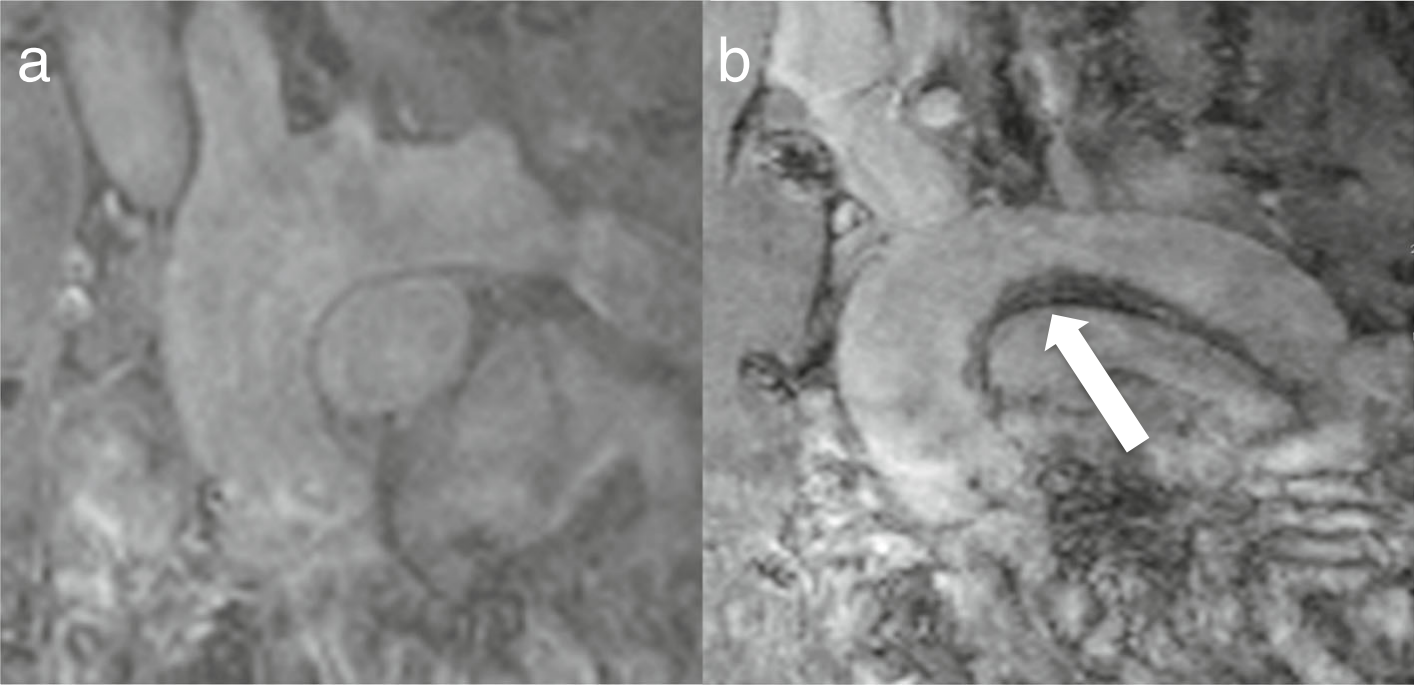
Aortic arch MPIO-αVCAM-1 binding in an uremic ApoE^−/−^ mice. Telediastolic T2*-weighted MR image acquired before (**a**) and after (**b**) the intravenous injection of 200 μL microparticles of iron oxide (MPIO) targeted against vascular cell adhesion molecule-1 (VCAM-1). White arrow, MR signal void in aortic arch is due to MPIO-αVCAM-1 binding

After intravenous MPIO-αVCAM-1 injection, 4/5 (80%) Ur mice (Fig. 5) and 1/4 (25%) NUr mice demonstrated a MR signal void in the aortic wall, whereas images were normal in all control mice (Table 3). There was a significant impact of animal group on MPIO-αVCAM-1 binding (global *p* value = 0.01), and post hoc analysis showed a significant difference between Ur and Ctl mice (*p* = 0.003). Signal void was mostly located within the aortic root. The localizations of MPIO-αVCAM-1 binding is summarized in Table 3.

**Table 3:** Contingency table for MPIO-αVCAM-1 binding localization using T2*-weighted MR images

Group of mice	Ur	NUr	Ctl
Aortic root (i)	4	1	0
Ascending aorta (ii)	1	1	0
Aorta braciocephalic trunk level (iii)	1	0	0
Brachiocephalic trunk (iv)	1	0	0
Aortic arch (v)	1	0	0
Total of animals	4/5	1/4	0/5

The site of binding is noted for each animal. An animal may have more than one site of binding. (i) The aortic root, (ii) the ascending aorta, (iii) the aorta at the level of the brachiocephalic trunk, (iv) the brachiocephalic trunk, and (v) the aortic arch. The groups of mice were uremic ApoE^−/−^ (Ur, *n* = 5), non-uremic ApoE^−/−^ (NUr, *n* = 5), and control mice (Ctl, *n* = 5)

Morphological MR imaging demonstrated a vascular dilation involving the aorta at the level of the brachiocephalic trunk, the brachiocephalic trunk itself, and the aortic arch in Ur compared to NUr and Ctl mice. The indexed vascular diameters were as follows: aorta at the level of the brachiocephalic trunk, 80.23 ± 3.50 μm/g (Ur, *n* = 4) vs. 67.62 ± 3.62 (NUr, *n* = 4) and 66.10 ± 2.94 (Ctl, *n* = 5, global *p* value < 0.05); brachiocephalic trunk, 36.36 ± 1.39 (Ur, *n* = 4) vs. 30.80 ± 0.65 (NUr, *n* = 4) and 31.30 ± 1.37 (Ctl, *n* = 5, global *p* value < 0.05); aortic arch, 70.49 ± 2.80 (Ur, *n* = 4) vs. 57.99 ± 2.92 (NUr, *n* = 4) and 55.70 ± 1.74 (Ctl, *n* = 5, global *p* value < 0.05); aortic root, 76.23 ± 7.62 μm/g (Ur, *n* = 4) vs. 66.69 ± 3.80 (NUr, *n* = 4) and 70.86 ± 1.51 (Ctl, *n* = 5, NS); and ascending aorta, 66.93 ± 3.96 μm/g (Ur, *n* = 4) vs. 60.30 ± 2.44 (NUr, *n* = 4) and 59.36 ± 3.58 (Ctl, *n* = 5, NS).

In addition, we analyzed the results of imaging at 16 weeks (positive vs. negative) with respect to the animal group (Ur, NUr, Ctl) and to the molecular imaging modality (PET and MPIO-αVCAM-1 MRI). The logistic regression demonstrated a significant effect of the animal group on the imaging results (*p* < 0.001), independent of the imaging modality (*p* = 0.99, ns). The odds ratios for positive imaging results were significant for Ur vs. NUr (*p* = 0.01) and Ur vs. Ctl (*p* < 0.001) but not for NUr vs. Ctl (*p* = 0.99, ns).

### Western blotting

**Fig. 6:**
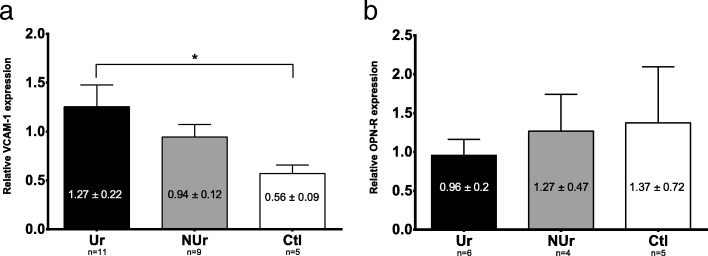
VCAM-1 (**a**) and OPN-R (**b**) aortic protein expression. The protein expression was normalized for each animal by GAPDH protein expression. **a** There was a significant increase of VCAM-1 protein expression in uremic ApoE^−/−^ (Ur, *n* = 11) compared to control Bl6 mice (Ctl, *n* = 5, respectively, *p < 0.05). **b** The decrease of OPN-R protein expression in Ur (*n* = 6) compared to NUr and Ctl mice (*n* = 4 and *n* = 5, respectively) was not significant. Data are expressed as mean ± SEM

The aortic expression of VCAM-1 protein was significantly different between animal groups (Ur 1.27 ± 0.22 AU, *n* = 11, NUr 0.94 ± 0.12, and Ctl 0.56 ± 0.09, global *p* value = 0.02), with a significant difference between uremic ApoE^−/−^ and control mice (*p* < 0.01, Fig. 6a). No difference was found between groups for aortic OPN-R protein expression (Fig. 6b).

## Discussion

In this study, a chronic renal failure (CRF) induced an increase of calcium plasma and tissular content in ApoE^−/−^ mice. This study demonstrated the feasibility of [18F]NaF PET to assess in vivo aortic mineralization in a mouse model of accelerated atherosclerosis. In addition, injected MPIO-αVCAM-1 MR imaging allowed the evaluation of active inflammation in this mouse model. Our results further demonstrated that the occurrence of mineralization and inflammation processes is associated with vascular remodeling in this animal model.

The CRF is associated with metabolic and endocrine abnormalities, including abnormal calcium and phosphate metabolism and an inflammatory syndrome. In our uremic ApoE^−/−^ mice, the increase of calcium plasma concentration was similar to previous results [13, 14]. Using a different model, Pulskens et al. also reported a significant increase of calcium plasma level observed 4 weeks after inducing a renal failure using adenine-enriched diet [15]. CRF involves an increased synthesis and secretion of parathyroid hormone (PTH) [13] that is an essential regulator of bone remodeling and calcium homeostasis [16], and results in increased vascular calcification [17]. As previously demonstrated [18], we confirmed using flame atomic absorption spectroscopy an increase of calcium content in aortic tissue of uremic ApoE^−/−^. On the other hand, the von Kossa staining found no difference between uremic ApoE^−/−^ and other animal groups. These findings are consistent with an early microcalcification process associating an increased calcium vascular content without macroscopic evidence of calcifications. The calcium/phosphate ratio and the vitamin D in the dietary regimen are important factors to observe macrocalcifications within the aortic wall. Using both a high calcium/phosphate ratio (1.6) and a high vitamin D diet (1540 IU/kg), Massy et al. [13] demonstrated macrocalcifications that we and others did not report when using a similar and lower calcium/phosphate ratio (1.3) and vitamin D intake (600–1000 IU/kg) [14].

Irkle et al. [19] demonstrated that [18F]NaF adsorbs to calcified deposits within plaque with a high affinity and that [18F]NaF PET/CT imaging can distinguish between areas of macro- and microcalcification. However, in vivo preclinical [18F]NaF PET/CT remains challenging and poorly investigated for the assessment of vascular mineralization process. In the present study, PET data were reconstructed using 3D-OSEM with 8 iterations and a zoom factor 2. Previous results demonstrated that these reconstruction parameters optimized spatial resolution (with a full width at half maximum (FWHM) below 1 mm) and allowed the investigation of active aortic mineralization [20] as confirmed by ex vivo measurements of aortic uptake using gamma counter. The present study confirms the feasibility of preclinical [18F]NaF PET for in vivo assessment of aortic microcalcifications.

ApoE^−/−^ mice develop various lesions of atherosclerosis throughout the arterial system, including foam cell lesions as early as 10 weeks when maintained on chow diet and fibrous plaques after 15 weeks [21]. In addition, uremia further accelerates both atherosclerotic lesions and arterial calcification in ApoE-deficient mice [13]. Our results are consistent with these latter studies. Although it was found in both uremic and non-uremic ApoE^−/−^ mice aged 12 weeks old, an active vascular mineralization process was demonstrated only in uremic ApoE^−/−^ mice aged 16 weeks old, suggesting that the CRF induced a sustained process of mineralization. Furthermore, the decrease in [18F]NaF uptake at 16 weeks old in animals with positive von Kossa staining emphasized that molecular [18F]NaF PET imaging mainly targets the micro- rather than macrocalcifications as previously suggested by ex vivo experiments [19]. Similar results were observed in patients showing positive [18F]NaF PET in regions with either no computed tomography (CT) detectable calcification or small foci of spotty calcification whereas macrocalcification remains the main target of CT [22]. Since atherosclerosis is a diffuse process involving the entire arterial system, global [18F]NaF uptake in the entire heart mesured as evidence for coronary artery atherosclerosis was significantly correlated to aortic uptake and may provide valuable information about the extent of the mineralization process as previously suggested [23].

Active inflammation is an early phenomenon in atherosclerosis [21]. Endothelial activation, a condition associated with most forms of cardiovascular diseases, involves phenotypic changes of the endothelial cell surface that allow leukocyte adherence and diapedesis to injured tissues. Vascular cell adhesion molecule-1 (VCAM-1) is not expressed by quiescent endothelial cells, and the measurement of soluble VCAM-1 is commonly used as a biomarker endothelial activation [8]. Preclinical MR imaging using MPIO targeted against VCAM-1 (MPIO-αVCAM-1) has been shown to demonstrate acute and chronic endothelial activation in various clinically relevant contexts in mice, including chronic renal failure [9]. In the present study, endothelial activation was detectable as a signal void using MPIO-αVCAM-1 T2* MR imaging in 80% of uremic mice and in 25% of non-uremic, but was undetectable in control mice. In addition, Western blotting corroborated increased aortic VCAM-1 expression in uremic mice.

Early results previously documented an increased VCAM-1 mRNA expression in aortas from uremic ApoE^−/−^ mice [14]. Nahrendorf et al. [24] developed VCAM-1-targeted nanoparticles that undergo internalization in cells expressing VCAM-1. Using this VINP (VCAM-1 internalizing nanoparticle), they demonstrated the feasibility of non-invasively imaging of VCAM-1 expression in atherosclerosis ApoE^−/−^ mice. They also confirmed early atherosclerotic lesions in juvenile ApoE^−/−^ mice (age, 9 weeks) on a high-cholesterol diet. However, internalized VINP colocalized with VCAM-1 in both endothelial cells and macrophages. Ultrasmall superparamagnetic particles of iron oxide (USPIO) are prone to unspecific extravasation due to their small size and have a lower relaxivity that requires high local concentrations to be detectable [25, 26]. MPIO-αVCAM-1 combines the larger size of micron-sized particles of iron oxide that prevents unspecific extravasation to a formulation targeted against VCAM-1 that ensures a high sensitivity mapping of endothelial activation. This technique has already been validated in various experimental models of acute or chronic endothelial activation [9, 27]. In addition to VCAM-1 mapping showing endothelial activation in the aortic arch and the brachiocephalic trunk, we also found a significant dilation of these segments. These enlarged segments match the sites of predilection for atherosclerotic lesion development [21], suggesting an association between endothelial inflammation and structural vascular remodeling.

## Conclusions

In this study, molecular imaging allowed in vivo characterization of the early phase of atherosclerosis. [18F]NaF PET showed early and sustained vascular mineralization in uremic ApoE^−/−^ mice, a model of accelerated atherosclerosis. In the same model, MPIO-αVCAM-1 MR imaging demonstrated aortic endothelial activation, predominantly in segments with vascular remodeling. In vivo dual assessment of vascular mineralization and endothelial activation opens up new perspectives for future evaluation of the dynamic process of atherosclerosis and new treatment strategies.

## Data Availability

The datasets used and/or analyzed during the current study are available from the corresponding author on reasonable request.

## Abbreviations

ApoE: Apolipoprotein
BMP: Bone morphogenetic proteins
CRF: Chronic renal failure
Ctl: Control C57Bl/6 J
FAAS: Flame atomic absoprtion spectroscopy
FDG: Fluorodeoxyglucose
ICAM: Intracellular adhesion molecular
MPIO: Micron-sized particles of iron oxide
MR: Magnetic resonance
NaF: Sodium fluoride
NUr: Non-uremic
OSEM: Ordered subset expectation maximization
PET: Positron emission tomography
Ur: Uremic
USPIO: Superparamagnetic particles of iron oxide
VCAM: Vascular cell adhesion molecule
VINP: VCAM-1 internalizing nanoparticle
VSMC: Vascular smooth muscle cell
